# The Bradford Royal Infirmary Project

**Published:** 1914-02-14

**Authors:** 


### THE BRADFORD ROYAL INFIRMARY PROJECT.

A big project, and one which is institutionally full of novelty and interest, has been set on foot in Bradford in connection with the rebuilding of the Royal Infirmary there. Some four or five years ago it was decided that the existing Infirmary must be rebuilt on a larger scale, and that a fresh site must be selected for the purpose. At present the Infirmary accommodates 210 beds, but the pressure on them is such that a hospital of nearly 400 was decided upon. In 1909 an admirable site was secured, and an appeal opened for the funds necessary to carry out the selected design, which was by Mr. W. A. Pite, the architect of the new King's College Hospital. The estimated cost was £220,000, towards which £58,000 was secured in the first year. Since then, however, the scheme has languished, for only £80,000 is in hand now, with which sum the governors have not felt justified in beginning to build.

Last summer Alderman Pickles, a Labour member of the City Council, drew attention to the City's urgent need of more hospital accommodation; and the result of this has been that a joint committee, representing in equal numbers the City Council, the Insurance Committee, and the Infirmary management, met and discussed the whole situation. The upshot is a scheme whereby the Corporation will acquire the whole of the present buildings and site of the Bradford Royal Infirmary for £100,000, subject to Local Government Board sanction. The property is to be valued; if the valuation comes to less than this sum the Corporation will nevertheless make it up to the six figures; if to more, the Corporation (apparently) will pay the total so fixed by the valuer. The buildings are to be utilised as offices and premises for various civic departments.

So far, so good. Manchester has set an example in the same direction by making a grant to the Royal Infirmary Building Fund in the past. The *quid pro quo*, however, is a clause in the agreement stipulating that the Bradford Corporation shall henceforth have representation on the board of management to the extent of one-fourth of its total membership. This provision would, of course, alter the constitution of the Infirmary in a very radical and fundamental manner. If the old site and buildings are honestly worth £100,000 to the Corporation, we see no reason for the stipulation; if they are not, then clearly this may prove a stride towards the complete municipalisation of the voluntary hospitals of Bradford (for it is stated that the Corporation desire to see the other hospitals—that is, the Bradford Children's Hospital and the Royal Eye and Ear Hospital—associated with the scheme). The board of the Infirmary does, it is true, publicly express the conviction that the purely voluntary nature of the work there will be safeguarded by the proposed scheme; but it is a little difficult to understand the basis of this confidence. It is noteworthy also that the Labour Party seems to have played a conspicuous part in the negotiations—another hint that municipal control and management may be what is aimed at. The people of Bradford may be wishful to consider a proposal of such magnitude very carefully before it is carried out; and there is much to be said in favour of a direct poll of the ratepayers before it is definitely enforced. The scheme has, however, been passed by the City Council without any such referendum.

